# Activation of CXCL6/CXCR1/2 Axis Promotes the Growth and Metastasis of Osteosarcoma Cells *in vitro* and *in vivo*

**DOI:** 10.3389/fphar.2019.00307

**Published:** 2019-03-28

**Authors:** Guangchen Liu, Liping An, Hongmei Zhang, Peige Du, Yu Sheng

**Affiliations:** ^1^Department of Traumatic Orthopedics, The First Hospital of Jilin University, Changchun, China; ^2^College of Pharmacy, Beihua University, Jilin, China; ^3^Department of Pharmacy, The First Hospital of Jilin University, Changchun, China

**Keywords:** osteosarcoma, CXCL6/CXCR1/2 axis, metastasis, PI3K/AKT, β-catenin

## Abstract

Osteosarcoma (OS) is a malignant primary bone tumor with high metastatic rate. C-X-C motif chemokine ligand 6 (CXCL6) and its receptor C-X-C motif chemokine receptor 1/2 (CXCR1/2) have been found to participate in the process of carcinogenesis. In this study, we evaluated the role of CXCL6/CXCR1/2 axis in proliferation and metastasis of OS cells. According to our results, the mRNA and protein expressions of CXCL6, CXCR1, and CXCR2 in multiple OS cell lines were determined. Treatment with exogenous CXCL6 for more than 72 h significantly promoted the proliferation of OS cells. Blocking the effect of endogenous CXCL6 restrained the migration, invasion and epithelial-mesenchymal transition (EMT) as evidenced by increased E-cadherin level, decreased N-cadherin and Snail levels in OS cells. On the contrary, exogenous CXCL6 administration enhanced the migration and invasive abilities of OS cells. Moreover, silencing of CXCR1/2 suppressed migration, invasion and EMT of OS cells with or without treatment with exogenous CXCL6. In addition, exogenous CXCL6 promoted the activation of PI3K/AKT and β-catenin signaling pathways, which could be repressed by CXCR2 knockdown. Inactivation of PI3K/AKT or β-catenin pathway by specific inhibitors effectively suppressed CXCL6-induced migration, invasion and EMT of OS cells. Finally, overexpression of CXCL6 significantly contributed to tumor growth, pulmonary metastasis and activation of PI3K/AKT and β-catenin pathways in nude mice *in vivo*, which were repressed by treatment with CXCR2 antagonist. Our results suggest that CXCL6/CXCR1/2 axis promotes the proliferation and metastasis of OS cells.

## Introduction

Osteosarcoma (OS) is one of common malignant primary bone tumors, which frequently occurs in adolescents or children and accounts for about 2.4% of all tumors in children (Ottaviani and Jaffe, 2009). At present, surgery combination with chemotherapy is a widely used treatment for OS. A great deal of patients with OS may suffer distant metastasis, and the prognosis is poor (Tan et al., 2009). Until the mid-1980s, the 5-year survival of OS had been increased from 20 to 70% (Chou and Gorlick, 2006). However, over the past decades, the 5-year survival of OS has not been further improved. Therefore, searching novel effective therapeutic targets for OS is of great significance.

Cytokines of the C-X-C motif chemokine ligand (CXCL) family serve different functions depending on the presence or absence of the amino acid triplet ELR in their protein sequence. In these regards, ELR+CXCL presents proangiogenic properties, whereas ELR-CXCL has antiangiogenic properties (Grepin et al., 2014). CXCL6, also known as Granulocyte Chemotactic Protein 2 (GCP2), was firstly found in MG-63, one of OS cell lines, by Proost et al. (1993). CXCL6 is one of ELR+CXCLs. Similar to CXCL8, also an ELR+CXCL, CXCL6 regulates its downstream pathways via binding with C-X-C motif chemokine receptor 1 (CXCR1) and C-X-C motif chemokine receptor 2 (CXCR2; Wolf et al., 1998). Previous research has confirmed the role of CXCL8/CXCR axis in the occurrence and development of OS (Du et al., 2018). It is noteworthy that the CXCL6 level in serum of OS patients has been found to be up-regulated and recombinant CXCL6 could promote the proliferation of OS cells (Li et al., 2011). It is widely acknowledged that CXCL6 promotes the growth and metastasis of various cancers, including non-small cell lung cancer (Li et al., 2018), colon cancer (Ma et al., 2017), and melanoma (Verbeke et al., 2011). The angiogenesis-promoting effect of CXCL6 on tumorigenesis and metastasis has also been confirmed (Ma et al., 2017). However, the role of CXCL6/CXCR1/2 axis in OS has not been determined. Based on the above research background, we speculate that CXCL6/CXCR axis may play important roles in the progression of OS.

It has been reported that about 30–40% of OS patients may undergo metastasis, and their 5-year survival rates were reduced to only about 20% (Wu et al., 2009). So, investigating the mechanisms of invasion and metastasis of OS cells is quite necessary. Epithelial mesenchymal transition (EMT) is a biological process that epithelial cells transform into mesenchymal cells, which promotes the migration, invasion and distant metastasis of cancer cells. Growing evidence has demonstrated that inhibition of EMT effectively suppressed the migration and invasion of OS cells (Jin et al., 2017; Zhang et al., 2017). It has been well documented that CXCR2 is closely related with EMT in various tumors (Sobolik et al., 2014; Zhou et al., 2015; Xiang et al., 2017). However, the role of CXCL6/CXCR1/2 axis in migration, invasion, and EMT of OS cells is not clear, which needs to be clarified.

In this study, we performed *in vitro* and *in vivo* experiments to investigate the role of CXCL6/CXCR1/2 axis in the growth and metastasis of OS and its related mechanisms.

## Materials and Methods

### Reagents

Recombinant human CXCL6 (rhCXCL6) was purchased from PeproTech (Rocky Hill, NJ, United States). Anti-CXCL6 antibody was obtained from Abcam (Cambridge, United Kingdom). LY294002 was purchased from Beyotime Biotechnology (Haimen, China). XAV939 was purchased from MedChemExpress (Monmouth Junction, NJ, United States).

### Cell Lines and Culture

MG63, 143B, SaOS-2, and U2OS cell lines were obtained from Zhong Qiao Xin Zhou Biotechnology Co., Ltd., (Shanghai, China). MG63, SaOS-2, and U2OS cells were cultured in Dulbecco’s Modified Eagle Medium (DMEM, BD, United States) supplemented with 10% fetal bovine serum (FBS, Hyclone, Logan, UT, United States). 143B cells were cultured in Eagle’s minimum essential medium (EMEM, Zhong Qiao Xin Zhou Biotechnology, Shanghai, China) supplemented with 10% FBS (Hyclone, Logan, UT, United States). All the cells were maintained at 37°C, under a 5.0% CO_2_ atmosphere.

### Transient Transfection and Lentivirus Infection

The siRNAs were synthesized by Genepharma Inc., (Shanghai, China). The sequences of CXCR2 and negative control (NC) siRNAs were as follows: si-CXCR2-1 (sense: 5′-CCGUCUACUCAUCCAAUGUUA-3′; anti-sense: 5′-UAACAUUGGAUGAGUAGACGG-3′), si-CXCR2-2 (sense: 5′-GGCAACAAUACAGCAAACUTT-3′; anti-sense: 5′-AGUUUGCUGUAUUGUUGCCTT-3′), NC (sense: 5′-UUCUCCGAACGUGUCACGUTT-3′; anti-sense: 5′-ACGUGACACGUUCGGAGAATT-3′). The OS cells were transiently transfected with the abovesiRNAs by Lipofectamine 2000 (Invitrogen, CA, United States) according to the instructions. The full length CXCL6 was synthesized and cloned into lentiviral vector. Then the 293T cells were transfected with lentiviral vector to produce lentivirus particles (Wanleibio, Shenyang, China). The U2OS cells were infected with CXCL6 or vector lentivirus particles and selected with puromycin (Solarbio, Beijing, China) to generate cells that stably express CXCL6.

### Cell Growth Assay

The growth of OS cells was assessed by cell counting kit-8 (CCK8). OS cells were seeded into 96-well plates (3 × 10^3^ cells/well). After treatment with 100 ng/ml rhCXCL6 for 0, 12, 24, 48, 72, and 96 h, cells were incubated with 10 μl of CCK-8 (Beyotime, Haimen, China) at 37°C for 1 h. The absorbance values at 450 nm were detected by a microplate reader (BioTek, Winooski, VT, United States).

### Enzyme Linked Immunosorbent Assay (ELISA)

The CXCL6 level in the supernatant fluid of cultured OS cells was determined by a CXCL6 ELISA kit (BOSTER, Wuhan, China) according to the manufacturer’s protocol. The concentration of CXCL6 was calculated according to the standard curve.

### Transwell Migration and Invasion Assays

The invasion and migration of OS cells were determined by Transwell chamber (Corning, NY, United States) coated with or without Matrigel (BD Biosciences, Franklin Lakes, NJ, United States), respectively. Briefly, the OS cells in 200 μl serum-free medium were added into the upper chambers, while 800 μl medium containing 30% FBS was added into the lower chambers. After receiving different treatments for 24 h, the non-invasive cells on the upper surface were erased. The cells on the lower surface were fixed in 4% paraformaldehyde, and stained with 0.4% crystal violet. Under a microscope (Olympus, Tokyo, Japan), the number of invasive or migrated cells was counted in five random fields and the images were taken at a magnification of 200×.

### Immunofluorescence Staining

The OS cells with different treatments were cultured in slides, fixed in 4% paraformaldehyde for 15 min, incubated with 0.1% Triton X-100 for 30 min, and blocked with 10% goat serum for 15 min. Then the slides were incubated with primary antibodies against E-cadherin (1:50, Proteintech, Rosemont, IL, United States), N-cadherin (1:50, Proteintech) at 4°C overnight. FITC-labeled anti-mouse IgG (1:200, Beyotime, Haimen, China) was added for 1 h at room temperature. Then the nuclei were counterstained with DAPI. The slides were observed under a fluorescence microscope (Olympus, Tokyo, Japan) at a magnification of 400×.

### Western Blot Assay

The OS cells or tumor tissues were homogenized and lysed by RIPA Lysis Buffer (Beyotime), supplemented with 1% PMSF (Beyotime). Nuclear and Cytoplasmic Protein Extraction Kit (Beyotime) was used to extract nuclear protein. Protein concentration was measured by Enhanced BCA Protein Assay Kit (Beyotime). The protein samples were separated on SDA-PAGE and transferred to PVDF membranes (Millipore, Massachusetts, United States). After being blocked with 5% skimmed milk for 1 h, the membranes were incubated with primary antibodies CXCR1 (1:400, BOSTER, Wuhan, China), CXCR2 (1:400, BOSTER), CXCL6 (1:1000, Abcam, Cambridge, United Kingdom), E-cadherin (1:2000, Proteintech, Rosemont, IL, United States), N-cadherin (1:1000, Proteintech), Snail (1:500, Proteintech), p-AKT (1:1000, Cell Signaling Technology, Trask Lane Danvers, MA, United States), AKT (1:1000, Cell Signaling Technology), β-catenin (1:1000, Cell Signaling Technology), MMP9 (1:500, Sangon Biotech, Shanghai, China), Histone H3 (1:1000, Abcam), and β-actin (1:500, Santacruz Biotechnology, Santa Cruz, CA, United States) at 4°C overnight. Then the membranes were incubated with HRP-labeled Goat Anti-Rabbit or Goat Anti-Mouse secondary antibodies (1:5000, Beyotime) for 45 min at 37°C and developed using the BeyoECL Plus (Beyotime). Protein bands were analyzed using Gel-Pro-Analyzer software (Media Cybernetics, Rockville, MD, United States).

### Real-Time PCR

Total RNA was isolated from OS cells using TRIpure total RNA isolation kit (BioTeke, Beijing, China) and reverse transcribed into cDNA by Super M-MLV reverse transcriptase (BioTeke). Real-Time PCR was carried out using 2 × Power Taq PCR MasterMix (BioTeke). The primers were synthesized by Sangon Biotech (Shanghai, China) and listed in Table 1. The mRNA expression was normalized to β-actin and calculated by 2^-ΔΔCT^ method.

**Table 1:** Oligonucleotide primer sets for real-time PCR.

Name	Sequence (5′–3′)	Length
CXCL6 F	ACTTGTTTACGCGTTACGCTGAG	23
CXCL6 R	TTCTTCAGGGAGGCTACCACTT	22
CXCR1 F	CCCTGCCCTTCTTCCTTTTC	20
CXCR1 R	ACACCATCCGCCATTTTGCT	20
CXCR2 F	TTCCGAAGGACCGTCTACTCA	21
CXCR2 R	AGGGTGAATCCGTAGCAGAAC	21
β-actin F	CTTAGTTGCGTTACACCCTTTCTTG	25
β-actin R	CTGTCACCTTCACCGTTCCAGTTT	24

### Gelatin Zymography

After different treatments, the culture medium of OS cells was collected. Then samples (60 μg) were subjected to 10% polyacrylamide gel containing gelatin. The gel was washed two times in washing buffer (2.5% Triton X-100, 50 mM Tris–HCl, 5 mM CaCl_2_, 1 μM ZnCl_2_, pH 7.6) for 40 min, and incubated in 50 mM Tris–HCl, 5 mM CaCl_2_, 1 μM ZnCl_2_, 0.02% Brij, 0.2 M NaCl at 37°C for 40 h. Then the gel was stained in 0.05% Coomassie Blue G-250 for 3 h and destained in 30% methanol and 10% acetic acid for 0.5 h, 20% methanol and 10% acetic acid for 1 h, 10% methanol and 5% acetic acid for 2 h. The clear bands were imaged and analyzed.

### *In vivo* Tumor Xenograft

U2OS cells (1 × 10^7^) that infected with lentivirus (LV-NC or LV-CXCL6) were subcutaneously injected into the nude mice (Beijing HFK Bioscience Co., Ltd., China). There were three experimental groups (*n* = 6 per group): LV-NC, LV-CXCL6, LV-CXCL6+SB225002. The mice in LV-CXCL6+SB225002 group were intraperitoneally injected with 200 μg SB225002 in DMSO three times a week. The mice in the other groups were injected with DMSO in the same volume. Tumor diameter and width were measured every 3 days after the formation of xenografted tumor. Twenty-three days after the inoculation of U2OS cells, the mice were sacrificed by euthanasia. The tumor tissues were collected and weighed. This study was carried out in accordance with the recommendations of international ethical guidelines and the National Institutes of Health Guide concerning the Care and Use of Laboratory Animals. The protocol was approved by the Institutional Animal Care and Use Committee of Beihua University Laboratory Animal Ethics Committee (approval number of the ethical certificate: 2017060207).

### *In vivo* Metastasis Assay

To evaluate pulmonary metastasis, 1 × 10^6^ U2OS cells were intravenously injected into nude mice via the tail vein. The grouping and treatment were performed as described above. Four weeks after the inoculation of U2OS cells, the mice were sacrificed and the lung metastatic nodules were observed.

### Immunohistochemical Staining

The tumor tissues were fixed in formaldehyde, embedded in paraffin and cut into 5-μm sections. Then the sections were deparaffinized and rehydrated in gradient ethanol solutions. After receiving heat-induced epitope retrieval, the sections were blocked in goat serum for 15 min at room temperature. Then the sections were incubated with PCNA (1:50, Santa Cruz, CA, United States) as the primary antibody at 4°C overnight. Biotin-labeled Goat Anti-Mouse IgG (1:200, Beyotime) was used. Then the sections were labeled with horseradish peroxidase, and visualized by 3,3′-Diaminobenzidine (DAB, Solarbio). The images were taken under a microscope at a magnification of 400×.

### Statistical Analysis

Data are expressed as mean ± SD. GraphPad Prism 5 software (La Jolla, CA, United States) was used to perform statistical analysis. Student’s *t*-test was used for comparison between two groups. One-way analysis of variance (ANOVA) followed by Bonferroni’s Multiple Comparison Test was performed when comparing several groups. *P* < 0.05 is considered to be statistically significant.

## Results

### Expressions of CXCL6, CXCR1, and CXCR2 in OS Cells

**FIGURE 1:**
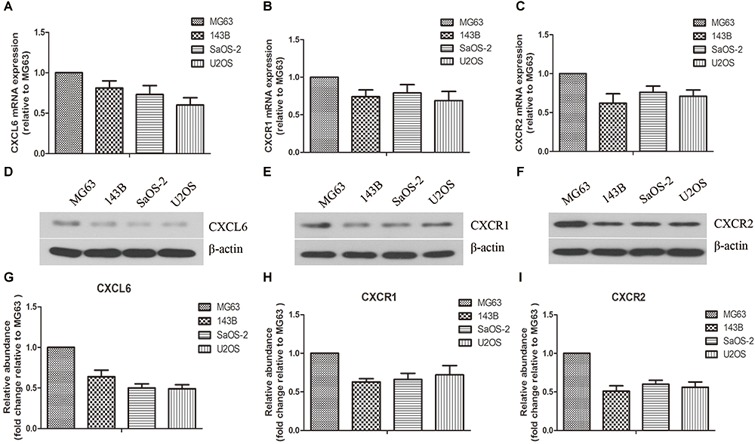
Expressions of CXCL6, CXCR1, and CXCR2 in osteosarcoma (OS) cells. The mRNA expressions of CXCL6 **(A)**, CXCR1 **(B)**, and CXCR2 **(C)** in multiple OS cell lines were evaluated by real-time PCR. The protein levels of CXCL6 **(D)**, CXCR1 **(E)**, and CXCR2 **(F)** in multiple OS cell lines were detected by western blot assay. **(G–I)** The protein quantification histograms were shown.

As shown in Figure 1A–C, the mRNA expressions of CXCL6, CXCR1 and CXCR2 in various OS cells (MG63, 143B, SaOS-2, U2OS) were determined. Among these OS cells, the mRNA expressions of CXCL6, CXCR1 and CXCR2 were highest in MG63. Similarly, the protein levels of CXCL6, CXCR1 and CXCR2, as detected by western blot, were highest in MG63 (Figure 1D–I).

### Effect of rhCXCL6 on the Proliferation of OS Cells

To assess the effect of rhCXCL6 on the proliferation of OS cells, CCK8 assay was performed. As illustrated in Supplementary Figures S1A–D, the proliferation of MG63, 143B, SaOS-2, and U2OS cells was significantly promoted after treatment with rhCXCL6 for more than 72 h. There was no significant difference within 48 h of rhCXCL6 treatment.

### Effect of Anti-CXCL6 Antibody on the Migration, Invasion and EMT of OS Cells

**FIGURE 2:**
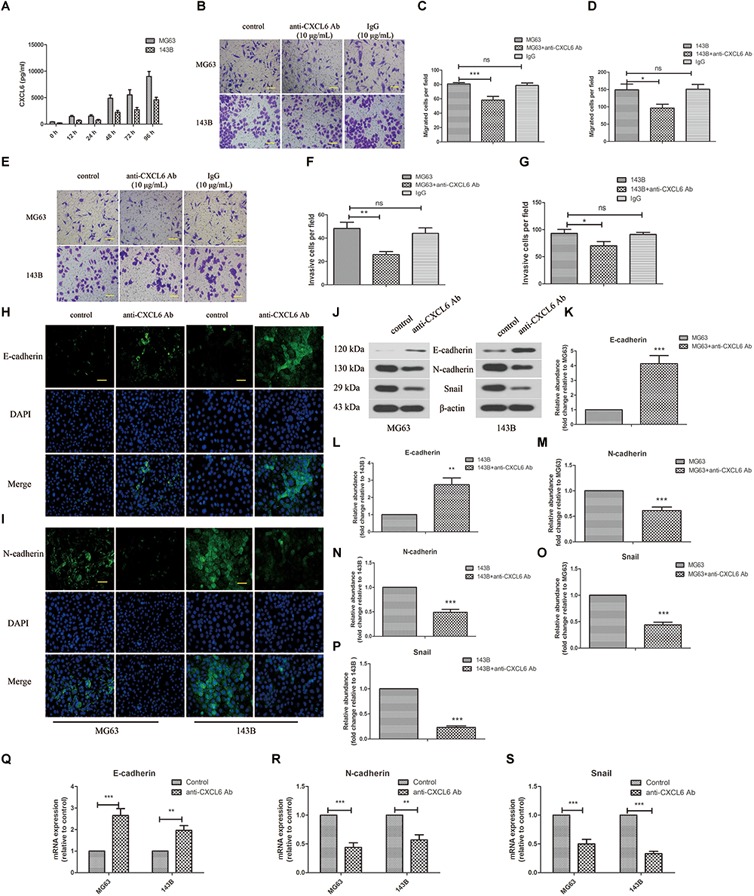
Anti-CXCL6 antibody inhibited the migration, invasion and EMT of OS cells. OS cells were cultured for 72 h, then incubated with anti-CXCL6 antibody (10 μg/mL) or a control antibody (IgG, 10 μg/mL) for another 24 h. **(A)** The level of CXCL6 in the supernatant fluid of cultured MG63 and 143B cells was detected by ELISA. **(B)** The migration of MG63 and 143B cells was evaluated by Transwell assay (no matrigel). Scal bar = 100 μm. **(C,D)** The number of migrated cells was shown. **(E)** The invasion of MG63 and 143B cells was assessed by Transwell assay (matrigel). Scal bar = 100 μm. **(F,G)** The number of invasive cells was shown. The expressions of E-cadherin **(H)** and N-cadherin **(I)** in MG63 and 143B cells were determined by immunofluorescence assay. Scal bar = 50 μm. **(J)** The protein levels of E-cadherin, N-cadherin, and Snail were evaluated by western blot assay. **(K–P)** The protein quantification histograms were shown. **(Q–S)** The mRNA expression of E-cadherin, N-cadherin, and Snail was detected by real-time PCR. ^∗^*P* < 0.05, ^∗∗^*P* < 0.01, ^∗∗∗^*P* < 0.001, versus the OS cell group.

The secretion level of CXCL6 by OS cells was detected by ELISA. As presented in Figure 2A, the level of CXCL6 secreted from MG63 and 143B cells was up-regulated gradually with time prolonging and the increase in the number of cells. To block the function of secreted CXCL6, 10 μg/ml anti-CXCL6 antibody was added to MG63 and 143B cells. To demonstrate the specificity of anti-CXCL6 antibody, the same amount of a control antibody (IgG) was used. As shown in Figure 2B–D, after treatment with anti-CXCL6 antibody for 24 h, the migration ability of MG63 and 143B cells was restrained. Moreover, the invasive ability of MG63 and 143B cells was also inhibited by incubation with anti-CXCL6 antibody for 24 h (Figure 2E–G). However, incubation with IgG did not affect the migration and invasion of MG63 and 143B cells. As shown in Figure 2H,I, the immunofluorescence staining of E-cadherin was enhanced, while N-cadherin was weakened in MG63 and 143B cells that received anti-CXCL6 antibody treatment. In addition, anti-CXCL6 antibody treatment resulted in increased protein level of E-cadherin, and decreased levels of N-cadherin and Snail in MG63 and 143B cells (Figure 2J–P). The mRNA expression of E-cadherin was increased, while N-cadherin and Snail were decreased after incubation with anti-CXCL6 antibody (Figure 2Q–S). These results indicated that blocking of endogenous CXCL6 function significantly restrained migration, invasion and EMT of MG63 and 143B cells.

### Effect of rhCXCL6 on the Migration and Invasion of OS Cells

**FIGURE 3:**
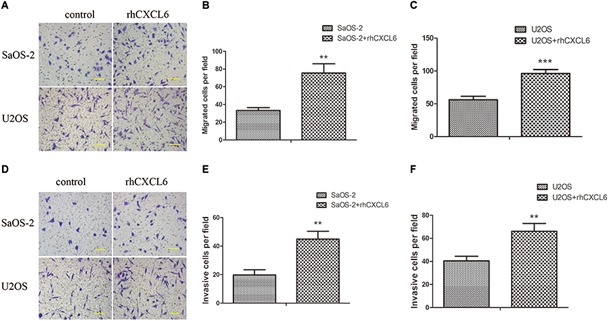
Recombinant human (rh) CXCL6 facilitated the migration and invasion of OS cells. OS cells were treated with 100 ng/ml rhCXCL6 for 24 h. **(A)** The migration of SaOS-2 and U2OS cells was detected by Transwell assay (no matrigel). Scal bar = 100 μm. **(B,C)** The number of migrated cells was shown. **(D)** The invasion of SaOS-2 and U2OS cells was determined by Transwell assay (matrigel). Scal bar = 100 μm. **(E,F)** The number of invasive cells was shown. ^∗∗^*P* < 0.01, ^∗∗∗^*P* < 0.001, versus the OS cell group.

Furthermore, we investigated the role of exogenous CXCL6 in the migration and invasion of OS cells. Since blocking the function of endogenous CXCL6 could inhibit the migration and invasion of OS cells, as expected, the migration ability of SaOS-2 and U2OS cells was strikingly improved by treatment with rhCXCL6 for 24 h (Figure 3A–C). Consistently, rhCXCL6 treatment significantly promoted the invasion of SaOS-2 and U2OS cells (Figure 3D–F).

### CXCL6/CXCR1/2 Axis Contributed to Migration, Invasion, and EMT of OS Cells

**FIGURE 4:**
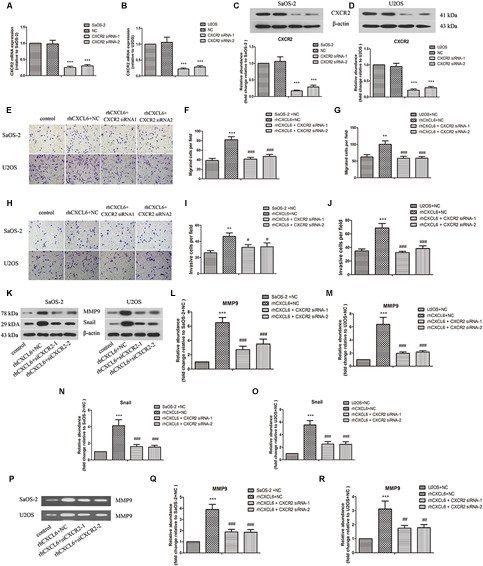
CXCL6/CXCR2 axis contributed to migration and invasion of OS cells. After transfection with siRNAs for 24 h, OS cells were treated with 100 ng/ml rhCXCL6 for 24 h. The mRNA expression of CXCR2 in SaOS-2 **(A)** and U2OS **(B)** cells was detected by real-time PCR. The protein expression of CXCR2 in SaOS-2 **(C)** and U2OS **(D)** cells was assessed by western blot assay. The protein quantification histograms were shown. **(E)** The migration of SaOS-2 and U2OS cells was detected by Transwell assay (no matrigel). Scal bar = 100 μm. **(F,G)** The number of migrated cells was shown. **(H)** The invasion of SaOS-2 and U2OS cells was determined by Transwell assay (matrigel). Scal bar = 100 μm. **(I,J)** The number of invasive cells was shown. **(K)** The protein levels of MMP9 and Snail in SaOS-2 and U2OS cells were detected by western blot assay. **(L–O)** The protein quantification histograms were shown. **(P)** MMP-9 activity in the supernatant fluid of cultured SaOS-2 and U2OS cells was determined by gelatin zymography method. **(Q,R)** The quantification histograms were shown. ^∗∗^*P* < 0.01, ^∗∗∗^*P* < 0.001, versus the SaOS-2+NC or U2OS+NC group.^#^*P* < 0.05, ^##^*P* < 0.01, ^###^*P* < 0.001, versus the rhCXCL6+NC group.

**FIGURE 5:**
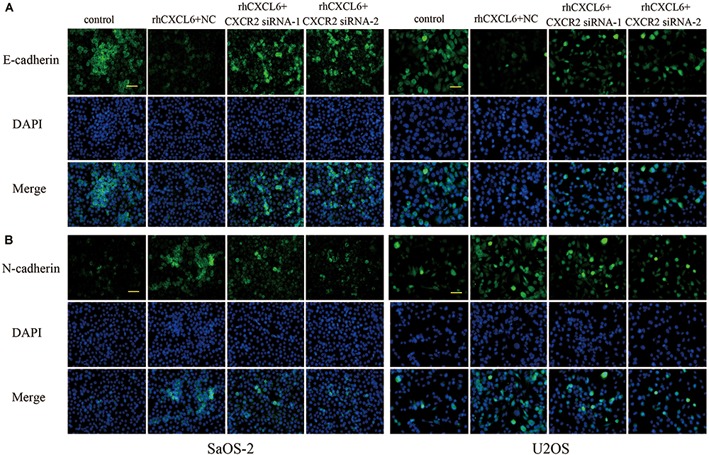
Effect of CXCL6/CXCR2 axis on E-cadherin and N-cadherin expressions. The expressions of E-cadherin **(A)** and N-cadherin **(B)** in SaOS-2 and U2OS cells with different treatments were determined by immunofluorescence assay. Scal bar = 50 μm.

CXCR1 and CXCR2 are recognized as receptors of CXCL6, so we further evaluated the role of CXCL6/CXCR1/2 axis in migration, invasion, and EMT of OS cells. To achieve this, the expression of CXCR1/2 was inhibited by transfection with CXCR1/2 siRNAs. As shown in Figure 4A–D and Supplementary Figures S2A–D, the mRNA and protein expressions of CXCR1/2 in SaOS-2 and U2OS cells were effectively repressed by transfection with CXCR1/2 siRNAs. Moreover, rhCXCL6-induced migration of SaOS-2 and U2OS cells was significantly inhibited by silencing of CXCR1/2 (Figure 4E–G and Supplementary Figures S3A–C). Likewise, the invasive ability that enhanced by rhCXCL6 treatment was effectively suppressed by CXCR1/2 siRNAs in SaOS-2 and U2OS cells (Figure 4H–J and Supplementary Figures S3D–F). In addition, rhCXCL6-induced increase in protein levels of MMP9 and Snail was evidently repressed by silencing of CXCR2 (Figure 4K–O). As detected by gelatin zymography and shown in Figure 4P–R, the activity of MMP9 in the supernatant fluid of cultured SaOS-2 and U2OS cells was distinctly increased by treatment with rhCXCL6, which could be restrained by silencing of CXCR2. As presented in Figure 5A,B silencing of CXCR2 significantly reversed rhCXCL6-induced decrease in E-cadherin expression, while increase in N-cadherin expression in SaOS-2 and U2OS cells. The above results suggested that CXCL6/CXCR1/2 axis was involved in the regulation of migration, invasion, and EMT of OS cells.

### Inhibition of CXCR1/2 Repressed the Migration and Invasion of OS Cells

As shown in Supplementary Figures S2E–J, inhibition of CXCR1/2 expression in SaOS-2 and U2OS cells by CXCR1/2 siRNAs repressed the migration and invasion of these cells accordingly.

### PI3K/AKT and β-Catenin Signaling Pathways Participated in the Regulation of Migration, Invasion and EMT by CXCL6/CXCR2 Axis in OS Cells

**FIGURE 6:**
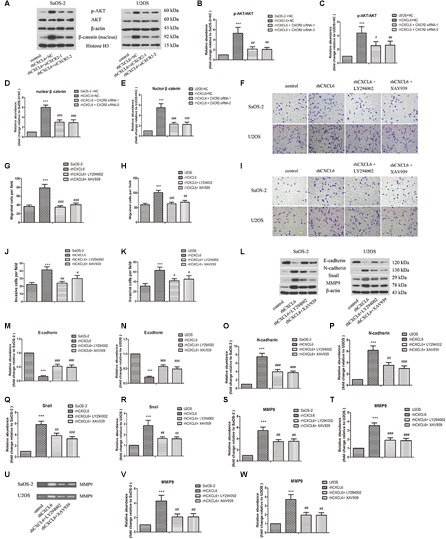
PI3K/AKT and β-catenin signaling pathways participated in the regulation of migration, invasion and EMT by CXCL6/CXCR2 axis in OS cells. After transfection with siRNAs for 24 h, OS cells were treated with 100 ng/ml rhCXCL6 for 24 h. **(A)** The protein levels of p-AKT, AKT, and nuclear β-catenin in SaOS-2 and U2OS cells were detected by western blot assay. β-actin and Histone H3 were used as loading controls. **(B–E)** The protein quantification histograms were shown. OS cells were pre-treated with 50 μM LY294002 or 10 μM XAV939 for 1 h, then treated with 100 ng/ml rhCXCL6 for 24 h. **(F)** The migration of SaOS-2 and U2OS cells was detected by Transwell assay (no matrigel). Scal bar = 100 μm. **(G,H)** The number of migrated cells was shown. **(I)** The invasion of SaOS-2 and U2OS cells was determined by Transwell assay (matrigel). Scal bar = 100 μm. **(J,K)** The number of invasive cells was shown. **(L)** The protein levels of E-cadherin, N-cadherin, Snail, and MMP9 in SaOS-2 and U2OS cells were detected by western blot assay. **(M–T)** The protein quantification histograms were shown. **(U)** MMP-9 activity in the supernatant fluid of cultured SaOS-2 and U2OS cells was assessed by gelatin zymography assay. **(V,W)** The quantification histograms were shown. ^∗∗∗^*P* < 0.001, versus the OS cell or OS cell +NC group.^#^*P* < 0.05, ^##^*P* < 0.01, ^###^*P* < 0.001, versus the rhCXCL6+NC or rhCXCL6 group.

To further evaluate the detailed mechanisms through which CXCL6/CXCR2 axis regulated malignant phenotype of OS cells, we focused on PI3K/AKT and β-catenin signaling pathways. As illustrated in Figure 6A–E, the ratio of p-AKT/AKT and nuclear β-catenin level was up-regulated by treatment with rhCXCL6, whereas knockdown of CXCR2 obviously inhibited the increased p-AKT/AKT ratio and nuclear β-catenin level. Furthermore, incubation with LY294002 (AKTpathway inhibitor) or XAV939 (β-catenin pathway inhibitor) strikingly suppressed rhCXCL6-induced increase in migration and invasion ability of SaOS-2 and U2OS cells (Figure 6F–K). Moreover, the decreased level of E-cadherin, increased levels of N-cadherin, Snail, and MMP9 induced by rhCXCL6 were evidently inhibited by administration of LY294002 or XAV939 (Figure 6L–T). As presented in Figure 6U–W, treatment with LY294002 or XAV939 significantly restrained the increased MMP9 activity induced by rhCXCL6.

### Effect of CXCL6/CXCR2 Axis on OS Tumor Growth and Pulmonary Metastasis *in vivo*

**FIGURE 7:**
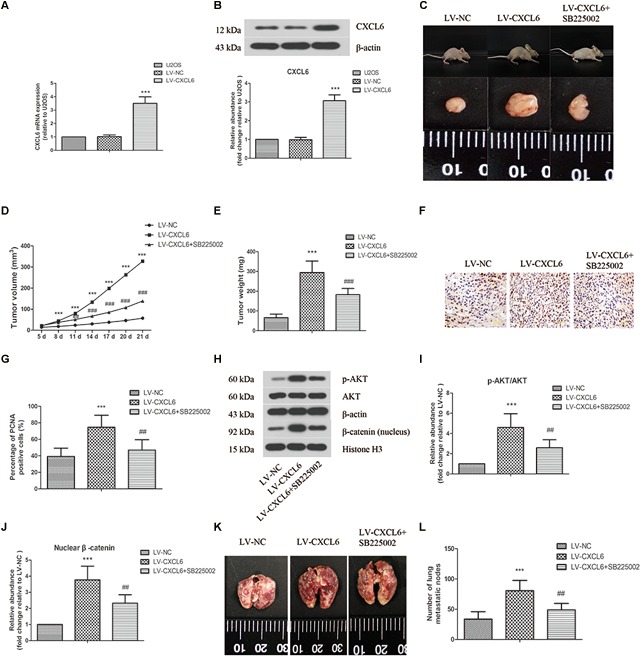
Effect of CXCL6/CXCR2 axis on OS tumor growth and pulmonary metastasis *in vivo*. U2OS cells were infected with lentivirus expressing CXCL6 or NC. The mRNA **(A)** and protein level **(B)** of CXCL6 in U2OS cells were determined by real-time PCR and western blot assay. **(C)** After the injection of LV-CXCL6 or LV-NC U2OS cells for 21 days, the representative images of nude mice and their removed xenograft tumors were shown. **(D)** The tumor volume was calculated and the tumor growth-curve was shown. **(E)** The weight of xenograft tumors was shown. **(F)** The expression of PCNA in tumor tissues was detected by immunohistochemical staining. Scal bar = 50 μm. **(G)** The percentage of PCNA positive cells were calculated and shown. **(H)** The protein levels of p-AKT, AKT, and nuclear β-catenin in tumor tissues were detected by western blot assay. β-actin and Histone H3 were used as loading controls. **(I,J)** The protein quantification histograms were shown. **(K)** Representative images of the lung of nude mice. **(L)** The number of lung metastatic nodes was quantified. ^∗∗∗^*P* < 0.001, versus the LV-NC group.^##^*P* < 0.01, ^###^*P* < 0.001, versus the LV-CXCL6 group.

As shown in Figure 7A,B lentivirus-mediated overexpression of CXCL6 in U2OS cells was confirmed by increased mRNA and protein levels of CXCL6. The volume and weight of xenograft tumors in CXCL6-overexpressed group were strikingly increased compared with those in NC group, which could be effectively restrained by treatment with SB225002, a potent and selective CXCR2 antagonist (Figure 7C–E). Moreover, the basal effect of SB225002 on the proliferation of OS cells was tested *in vitro*. As shown in Supplementary Figure 4, incubation with SB225002 significantly suppressed the proliferation of MG63, 143B, SaOS-2, and U2OS cells. As illustrated in Figure 7F,G there were more PCNA-positive cells in tumors formed by CXCL6-overexpressed cells, however, SB225002 treatment significantly suppressed PCNA expression. Moreover, the p-AKT/AKT ratio and nuclear β-catenin level in tumor tissues were enhanced in CXCL6-overexpressed group, whereas administration of SB225002 could reverse these changes (Figure 7H–J). As shown in Figure 7K,L the number of lung metastatic nodes, as assessed by tail vein injection of U2OS cells, was promoted in CXCL6-overexpressed group, which was evidently suppressed by SB225002 treatment.

## Discussion

Chemokines are a kind of secreted proteins that take part in multiple physiological and pathological processes, including immunity, body’s homeostatic regulation, oncogenesis, and so on (Baggiolini, 2001; Rot and Von Andrian, 2004). The pro-angiogenic ELR+CXCL family includes CXCL1, 2, 3, 5, 6, 7, and 8. A large body of research has suggested that these ELR+CXCLs promote the progression of cancer (Guo et al., 2017; Wang et al., 2017; Zhao et al., 2017; Xin et al., 2018). CXCL6 can be secreted from a variety of tumor cells, including OS cells, and plays crucial roles in the development of tumors (Gijsbers et al., 2005; Engl et al., 2006; Zhu et al., 2006). Like other ELR+CXCLs, CXCL6 was confirmed to have angiogenic properties in tumors (Van Coillie et al., 2001). It has been found that the plasma level of CXCL6 was up-regulated in OS patients, which associated with a poorer outcome (Li et al., 2011). However, the potential mechanisms of CXCL6 in the growth and metastasis of OS cells have not been determined. As OS cells secrete CXCL6 and express CXCR1 and CXCR2, we further evaluated the biological functions of CXCL6/CXCR1/2 axis in OS. Consistent with previous study, treatment with exogenous CXCL6 significantly promoted the proliferation of OS cells.

Next, we further investigated the effect of CXCL6 on metastasis of OS cells. Ma et al. reported that promoting CXCL6 secretion enhanced the metastatic potential of colon cancer (Ma et al., 2017). Previous study also demonstrated that inhibition of CXCL6 expression restrained the migration and invasion of hepatocellular carcinoma cells (Tian et al., 2014). These studies indicated that CXCL6 was involved in the metastasis of tumor cells. According to our results, blocking the function of endogenous CXCL6 obviously inhibited the migration and invasion of OS cells, while adding exogenous rhCXCL6 had the opposite effect. So, these results suggested that CXCL6 participated in the metastasis of OS cells.

In the process of EMT, the expression of epithelial cell marker E-cadherin is reduced, while the expression of mesenchymal marker N-cadherin is increased (Yang et al., 2016). Snail is confirmed to be a regulator of EMT, which can suppress E-cadherin expression in transcription level (Nieto, 2002). In addition, increased activity of MMP9 may result in decrease in E-cadherin level, impaired cell adhesion and enhanced cell motility via inducing EMT (Cowden Dahl et al., 2008). Cheng et al. suggested that CXCL8 promoted the progression of colorectal cancer by inducing EMT (Cheng et al., 2014). Inhibition of CXCL2 by short hairpin RNA suppressed the expressions of EMT markers in colorectal cancer cells (Chen M.C. et al., 2018). Like CXCL2 or CXCL8, we suspected that CXCL6 might also regulate EMT process in OS cells. In the present study, treatment with anti-CXCL6 antibody significantly increased E-cadherin level, while reduced N-cadherin and Snail levels. Moreover, rhCXCL6 administration had the opposite effect as we expected. These findings suggested that CXCL6 contributed to metastasis of OS cells via induction of EMT.

The ELR+CXC chemokines perform their biological functions through binding to CXCR1/2 receptors. Among them, only CXCL6, CXCL7 and CXCL8 bind to both CXCR1 and CXCR2, while the others bind to CXCR2 (Liu et al., 2016). Previous studies have demonstrated that CXCL8 and CXCL7 regulated the progression of various cancers via binding to CXCR1 and CXCR2 (Grepin et al., 2014; Liu et al., 2016). So, we further investigated whether CXCL6 regulated metastasis of OS cells via CXCR1/2. Inhibition of CXCR1/2 by SCH-527123 restrained cell proliferation, migration and invasion in melanoma (Shang and Li, 2018). The activation of CXCR1/Akt signaling pathway facilitated anoikis resistance and pulmonary metastasis of OS cells (Du et al., 2018). In addition, growing evidence has demonstrated that CXCR2 plays pivotal roles in the progression of various cancers (Ding et al., 2016; Maeda et al., 2017; Xiang et al., 2017). CXCR2 has been recognized as a predictor for the prognosis of gastric cancer patients, and also as a promising therapeutic target (Wang et al., 2015). However, it is not yet clear whether CXCR1/2 is involved in the malignant development of OS. According to our results, knockdown of CXCR2 strikingly repressed migration, invasion and EMT of OS cells with or without treatment with exogenous CXCL6. Therefore, CXCL6/CXCR1/2 axis participated in the metastasis of OS cells.

It has been well documented that Wnt/β-catenin and PI3K/AKT signaling pathways are aberrantly activated in multiple cancers, including OS (Park et al., 2015; Cui et al., 2018; Zhao et al., 2018). A large body of research indicated PI3K/AKT pathway participated in most malignant phenotypes of OS, and accelerated the progression of OS (Chen J. et al., 2018; Jin et al., 2018). Furthermore, PI3K/AKT pathway may restrain GSK3β activation that promotes β-catenin degradation, so PI3K/AKT facilitates Wnt/β-catenin pathway activation (Wu et al., 2012; Liu et al., 2013). In this study, our results showed that rhCXCL6 treatment promoted the activation of PI3K/AKT and Wnt/β-catenin pathways, which could be inhibited by silencing of CXCR2. In addition, inactivation of PI3K/AKT or Wnt/β-catenin pathway significantly restrained rhCXCL6-induced migration, invasion, and EMT of OS cells. So PI3K/AKT and Wnt/β-catenin pathways were involved in the regulatory mechanisms of CXCL6/CXCR2 axis in OS metastasis.

Finally, the above findings were further verified in nude mice *in vivo*. The results suggested that overexpression of CXCL6 contributed to tumor growth and pulmonary metastasis via activating PI3K/AKT and Wnt/β-catenin pathways, which could be repressed by treatment with CXCR2 antagonist SB225002. Recently, increasing evidence indicated that the composition of tumor microenvironment contributes to the progression of cancer. The tumor microenvironment is composed of extracellular matrix and stromal cells, including fibroblasts, vessel cells, and inflammatory leukocytes (Raman et al., 2007). The chemokines secreted by tumor cells may regulate these cells (Raman et al., 2007). CXC chemokines and their receptors have been discovered to be involved in tumor-stromal interactions in tumor microenvironment (Sano et al., 2019). So, in this study, SB225002, as a CXCR2 antagonist, may also block the effect of CXCL6, CXCL8 on the communication between OS cells and tumor microenvironment, which needs to be evaluated in our future research.

## Conclusion

Taken together, the present study demonstrated that CXCL6/CXCR1/2 axis promoted the proliferation, migration, invasion, and EMT of OS cells via activating PI3K/AKT and Wnt/β-catenin pathways *in vitro* and *in vivo*.

## Data Availability

No datasets were generated or analyzed for this study.

## Author Contributions

PD and YS studied the concepts and design. GL, LA, and HZ performed experimental studies. GL, LA, and HZ contributed reagents, materials, and analysis tools. GL wrote the manuscript. PD and YS reviewed and edited the manuscript. YS acquired the funds and administered the project.

## Conflict of Interest Statement

The authors declare that the research was conducted in the absence of any commercial or financial relationships that could be construed as a potential conflict of interest.
