# Supplementary material for: Activation of CXCL6/CXCR1/2 Axis Promotes the Growth and Metastasis of Osteosarcoma Cells in vitro and in vivo
Source: Front Pharmacol. 2019 Mar 28;10:307. doi: 10.3389/fphar.2019.00307 (PMC6447780; doi:10.3389/fphar.2019.00307)
Supplement: Supplementary file 1 [file Table_1.DOC]

**Supplementary Figure legends**

**Supplementary Figure 1** Recombinant human (rh) CXCL6 promoted the proliferation of OS cells. OS cells were treated with 100 ng/ml rhCXCL6, and at the indicated time points, the proliferation of MG63 (A), 143B (B), SaOS-2 (C), and U2OS (D) cells was assessed by CCK8 assay. *P < 0.05, versus the OS cell group.

**Supplementary Figure 2** CXCL6/CXCR1 axis contributed to migration and invasion of OS cells. After transfection with siRNAs for 24 h, OS cells were treated with 100 ng/ml rhCXCL6 for 24 h. The mRNA expression of CXCR1 in SaOS-2 (A) and U2OS (B) cells was detected by real-time PCR. The protein expression of CXCR1 in SaOS-2 (C) and U2OS (D) cells was assessed by western blot assay. The protein quantification histograms were shown. (E) The migration of SaOS-2 and U2OS cells was detected by Transwell assay (no matrigel). Scal bar=100 μm. (F)&(G) The number of migrated cells was shown. (H) The invasion of SaOS-2 and U2OS cells was determined by Transwell assay (matrigel). Scal bar=100 μm. (I)&(J) The number of invasive cells was shown. ***P < 0.001, versus the NC or control group. ^###^P < 0.001, versus the rhCXCL6+NC group.

**Supplementary Figure 3** Inhibition of CXCR1/2 repressed the migration and invasion of OS cells. (A) The migration of SaOS-2 and U2OS cells was detected by Transwell assay (no matrigel). Scal bar=100 μm. (B)&(C) The number of migrated cells was shown. (D) The invasion of SaOS-2 and U2OS cells was determined by Transwell assay (matrigel). Scal bar=100 μm. (E)&(F) The number of invasive cells was shown. ***P < 0.001, versus the NC group.

**Supplementary Figure 4** SB225002 suppressed the proliferation of OS cells. OS cells were treated with 2.5 μM SB225002, and at the indicated time points, the proliferation of MG63 (A), 143B (B), SaOS-2 (C), and U2OS (D) cells was assessed by CCK8 assay. *P < 0.05, **P < 0.01, versus the OS cell group.
