# Supplementary figures and images for: Activation of CXCL6/CXCR1/2 Axis Promotes the Growth and Metastasis of Osteosarcoma Cells in vitro and in vivo
Source: Front Pharmacol. 2019 Mar 28;10:307. doi: 10.3389/fphar.2019.00307 (PMC6447780; doi:10.3389/fphar.2019.00307)

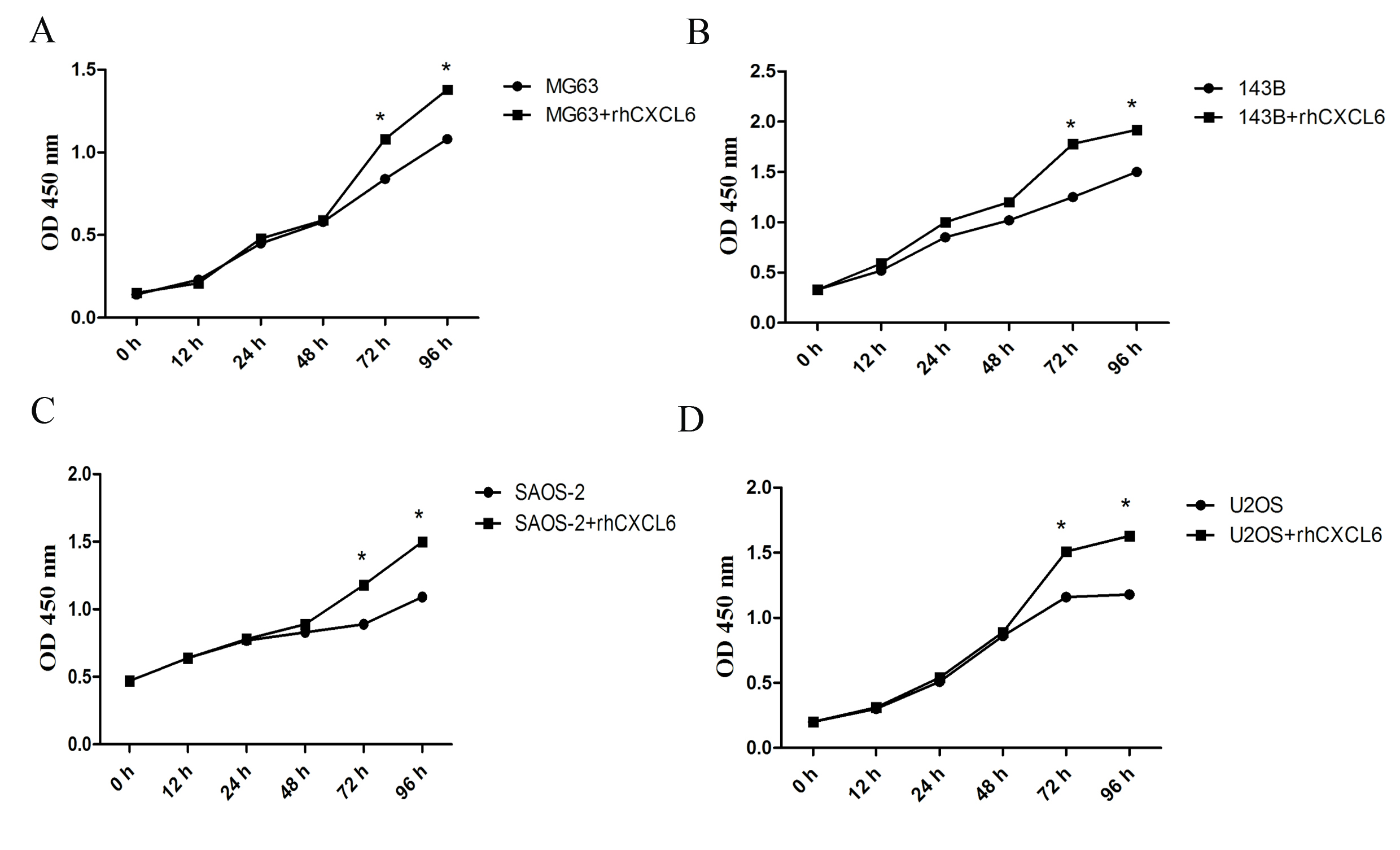

Supplement: Supplementary file 2 [file Image_1.JPEG]

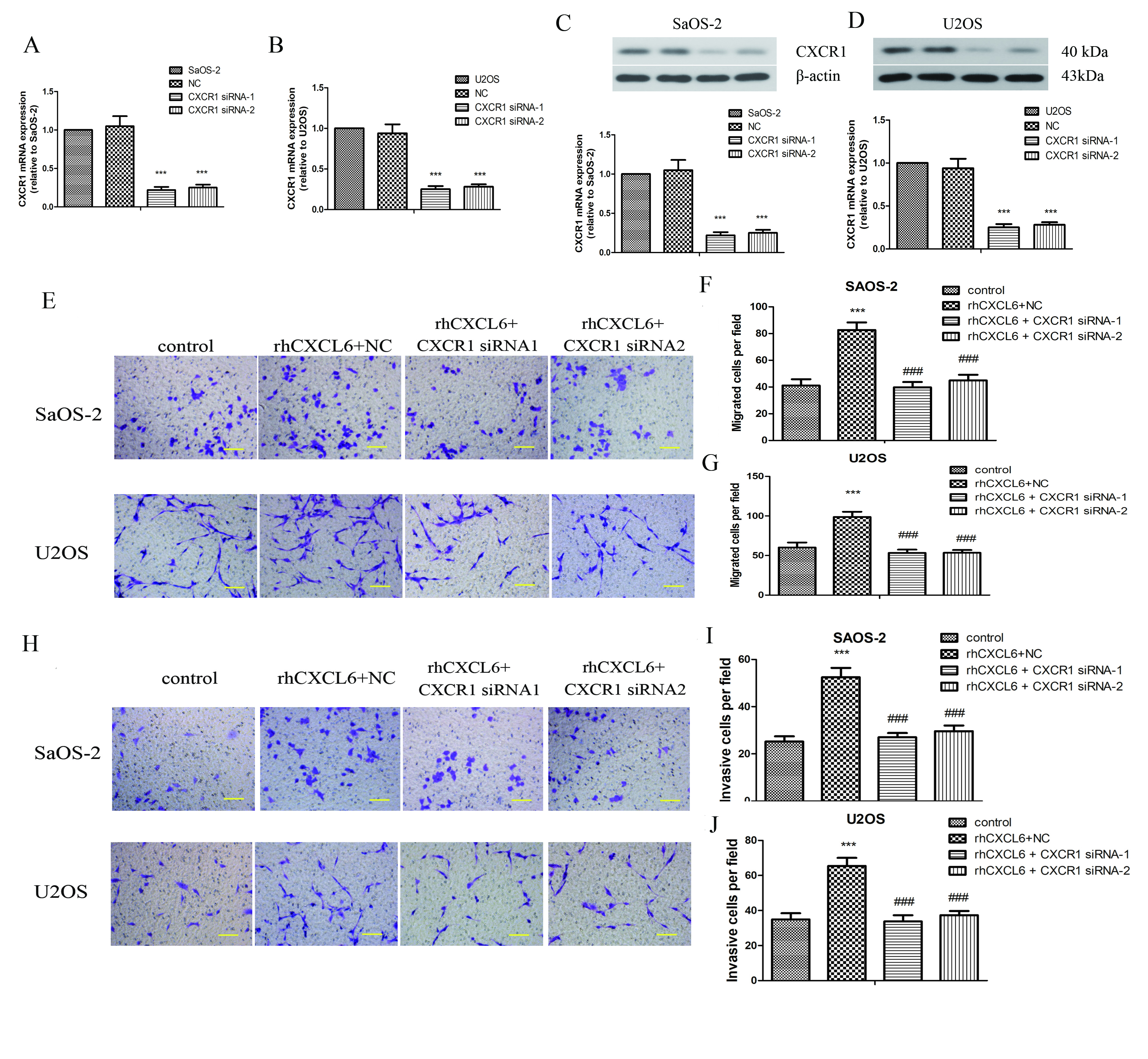

Supplement: Supplementary file 3 [file Image_2.JPEG]

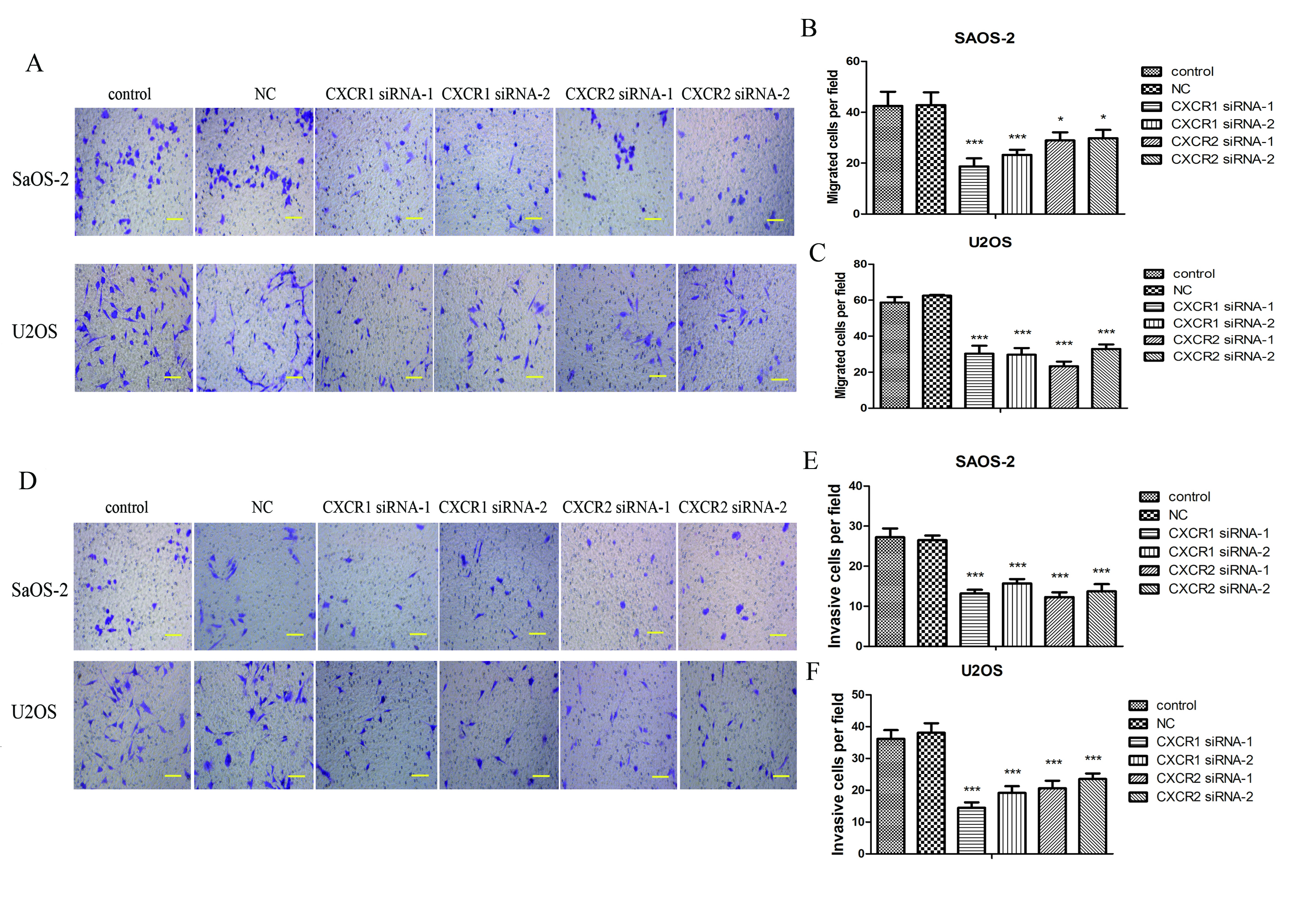

Supplement: Supplementary file 4 [file Image_3.JPEG]

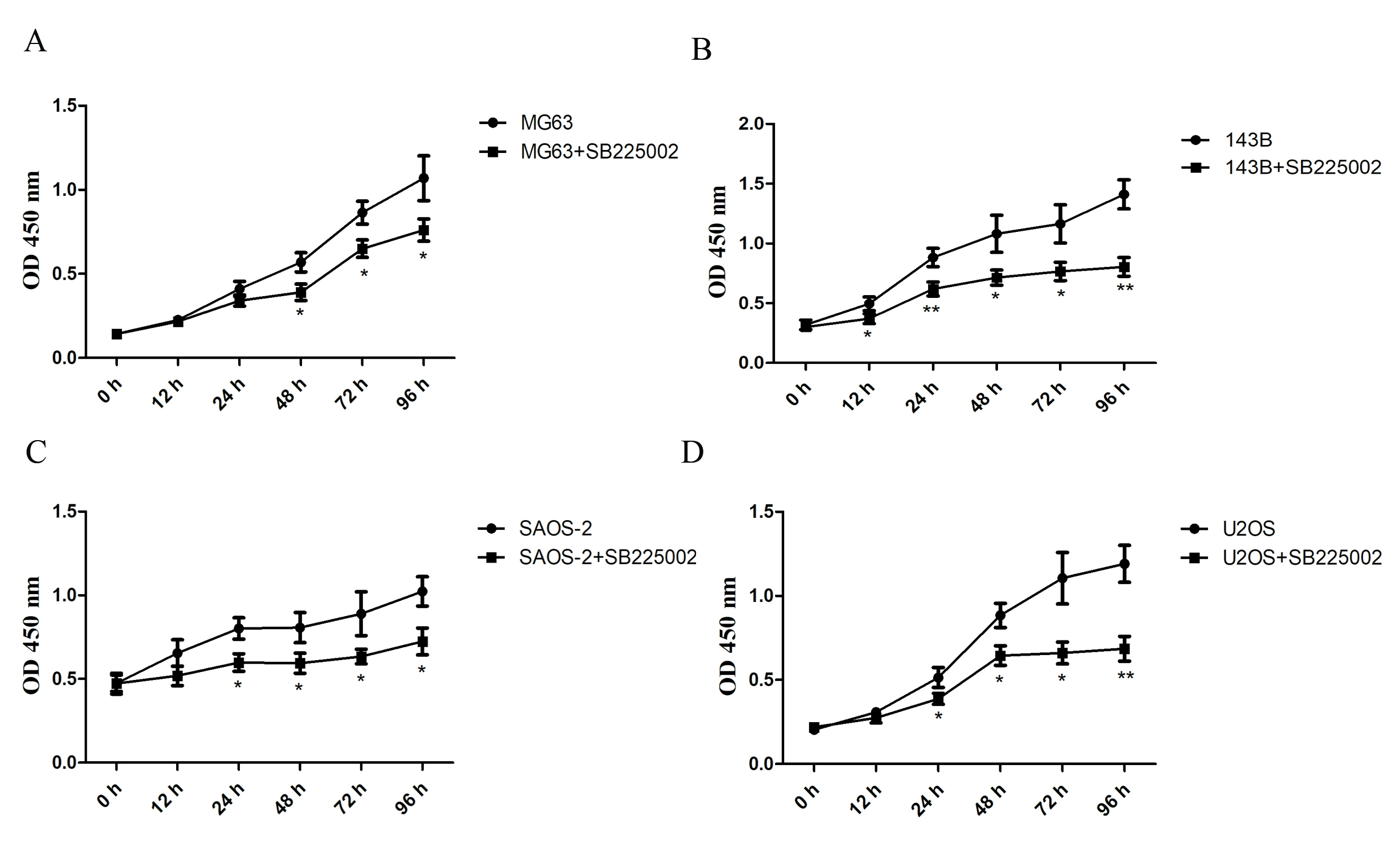

Supplement: Supplementary file 5 [file Image_4.JPEG]
